# Data for the narrative skills of Urdu-speaking preschoolers

**DOI:** 10.1016/j.dib.2021.106737

**Published:** 2021-01-11

**Authors:** Saboor Hamdani, Saira Ambreen

**Affiliations:** Centre for Clinical Psychology, University of the Punjab, Lahore, Pakistan

**Keywords:** Narrative skills, Personal narratives, Language development, Urdu

## Abstract

A total of 80 participants were recruited from three private middle SES schools of Rahim Yar Khan, Pakistan to explore the narrative skills development in Urdu-speaking preschoolers aged between 4-and-5 years. Data collection was completed using a two-stage sampling technique (convenience and purposive). After obtaining parental consent, the participants were screened for their intellectual functioning. Three personal narrative samples, using conversational maps, were collected from the participants who passed the screening. The narrative data were analysed on both macro and microstructural levels by using high point analysis and use of cohesions (conjunctive and referential), mean length of utterances in words (MLUw), the total number of words (TNW), and the number of different words (NDW). The data presented here include age and gender of participants, their scores on the verbal IQ test, the scores obtained on macro-and microstructural levels. The results based on differences and relationships of the data obtained are published somewhere else [1]. The data can be reused by comparing the obtained figures with the adult population, children from different age bands, children with different developmental disorders, and at cross-linguistic levels as narratives are said to be significantly influenced by cultures.

## Specifications Table

**Table:** 

Subject	Linguistics
Specific subject area	Narrative skills development and child language acquisition
Type of data	Excel file of the raw data
How data were acquired	Three narrative samples were collected from each participant individually by using an audio recorder.
Data format	Raw data file in .xlsx format.
Ethics Statement	The approval to conduct the research was obtained from the Departmental Doctoral Programme Committee (DDPC), Center for Clinical Psychology, The University of Punjab, Lahore. Permissions to use the instruments for data collection were taken from the respective authors. Moreover, permission to collect data was acquired from the concerned school authorities. Informed consent was taken from the parents of selected participants (with permission of school authorities), after providing them with details of the study. The children whose parents provided informed consent were recruited as participants. The parents were ensured about the confidentiality and right to withdraw any time on behalf of their children.
Parameters for data collection	The final data based on the personal narratives were obtained from the participants individually after they passed the screening assessing their intellectual functioning. Before the elicitation of the narratives, the children were given colors and coloring sheets. This was done to make them comfortable. The narratives were collected using different topics like birthday or visit to the zoo. If the child started to tell a narrative based on another topic of his/her interest, that was also included.
Description of data collection	Initially, the children were screened for intellectual functioning and the average time of screening per child was 10-15 minutes. Subsequently, the next day the narrative samples were collected from the children who passed the screening. Keeping in mind the comfort of children, the screening and narrative data collection were done on different days. The narrative samples were collected in separate available rooms in the school setting. Three narrative samples were collected from each child by using Conversational Maps [2]. All the narratives were collected in the Urdu language and were audiotaped.
Data source location	Three private schools of Rahim Yar Khan, Punjab, Pakistan
Data accessibility	The raw data file is with the article. If the readers want to gain some insight that how narrative samples were analysed and scored, the related research article has some examples in the section ‘Narrative Analyses’. If the readers need to access the related research article or/and narrative transcriptions for other research purposes they can contact authors in person.
Related research article	This data article is related to the following research article: Hamdani, S, Z., Arshad, T., Tarar, S., & Kausar, R. (2019). Personal Narrative Skills of Urdu Speaking Preschoolers. Narrative Inquiry. doi: https://doi.org/10.1075/ni.17063.ham

## Value of the Data

• To the best of our knowledge, these data that are based on the narrative samples of Urdu speaking preschoolers are the first one of their kind. The data will help to form a baseline for future studies in the related domains.
• All the researchers working in the field of language development not only from Pakistan where Urdu is the lingua franca but also from around the globe where the Urdu-speaking children reside as social ethnic minorities can benefit from this data.
• The presented data can be further utilized to design studies based on the comparison of these 4-and-5-year olds with Urdu-speaking adults and children from different age groups. These data are based on typically developing (TD) children, henceforth, can also be used for comparing the macro-and microstructural skills of TD children with different developmental disorders, to find out the deviated patterns. Moreover, this data can also be used for cross-linguistic comparisons.

## 1 Data Description

The attached excel file contains the demographic information of the participants including age and gender. In addition, it includes the verbal IQ scores, macrostructure scores analysed using High point analysis [2], and the scores obtained at the microstructural level of each participant. The scores based on different microstructure measures namely, the use of cohesive devices (conjunctions), MLUw, NWD, and TNW are also included in the data file.

## 2 Experimental Design, Materials and Methods

### 2.1 Research design

The between-group cross-sectional research design was employed to explore the patterns of personal narrative development in the Urdu-speaking preschoolers.

### 2.2 Participants, sampling, and demographics

Eighty Urdu-speaking children were recruited by using the two-stage sampling technique from three middle socioeconomic-status (SES) schools of Rahim Yar Khan, Pakistan. The stage one employed the convenience sampling technique meaning that the children whose parents were available and were willing to provide the consent of participation were included. Then in stage two by applying the purposive sampling technique, the children were screened for their intellectual functioning, and those who passed the screening were included. Slosson Intelligence Test, Revised (SIT-R3) [3] was administered for this purpose. This intelligence test was translated and adapted to Urdu by using MAPI guidelines [4]. SIT includes 187 items targeting cognitive domains of general information, vocabulary, comprehension, quantitative ability, similarities and differences, and auditory memory with overall test-retest reliability of .96. The total standard score for average and above average was 89–109 and 110–119, respectively. The participants who scored at least in the average range were included.

Based on the screening results, the final sample included two main groups with 40 children each, divided into groups based on their age, 4-year-olds *(M = 4.33, SD = .26),* and 5-year-olds *(M = 5.46, SD = .19).* There were 38 girls and 42 boys in the final sample. Additional information based on the acquisition of language developmental milestones was obtained from the mothers within a demographic form (constructed by researchers) attached to the consent form.

### 2.3 Narrative elicitation procedure

The personal narratives were elicited using Peterson and McCabe's [5] Conversational Map Elicitation Procedure [2]. For details please see the related research article.

### 2.4 Assessment measures

The narrative analyses were carried out both on macro and microstructural levels. For macrostructure, high point analysis by McCabe and Rollins [2] was used. The microstructure analysis included different in-depth linguistic measures like MLUw, NDW (40 words), NDW, TNW, and use of the cohesive device (for detail please refer to the related research article).

### 2.5 Statistical analysis

All the statistical analyses were conducted using SPSS v21.

## Declaration of Competing Interest

The authors declare that they have no known competing financial interests or personal relationships which have, or could be perceived to have, influenced the work reported in this article.

## Appendix Supplementary materials

Image, application 1
