# Corrigendum to “Data for the narrative skills of Urdu-speaking Preschoolers” [Data in Brief, 34 (2021) 106,737]

**DOI:** 10.1016/j.dib.2021.106911

**Published:** 2021-02-26

**Authors:** Saboor Hamdani, Saira Ambreen

**Affiliations:** a Department of Chinese and Bilingual Studies, The Hong Kong Polytechnic University, HKSAR; b Research Centre for Language, Cognition, and Neuroscience, The Hong Kong Polytechnic University, HKSAR; c Academic Unit of Human Communication, Development, and Information Sciences (CDIS), Faculty of Education, The University of Hong Kong, HKSAR; d Former Institute*,* Centre for Clinical Psychology, University of the Punjab, Lahore, Pakistan

The authors regret that the current affiliations were not mentioned in the original article.

The authors would like to apologise for any inconvenience caused.

